# Purification and Characterization of a Novel Thermostable Papain Inhibitor from *Moringa oleifera* with Antimicrobial and Anticoagulant Properties

**DOI:** 10.3390/pharmaceutics13040512

**Published:** 2021-04-08

**Authors:** Juliana Cotabarren, Santiago Claver, Juan Abreu Payrol, Javier Garcia-Pardo, Walter David Obregón

**Affiliations:** 1 Centro de Investigación de Proteínas Vegetales (CIPROVE), Departamento de Ciencias Biológicas, Facultad de Ciencias Exactas, Universidad Nacional de la Plata, Buenos Aires B1900, Argentina; sclaver@hotmail.com; 2 Escuela Latinoamericana de Medicina, La Habana 19108, Cuba; jabreu@bionaturasm.cu; 3 ECTI “Sierra Maestra”, Complejo Barlovento Ave 5ta. y 246, Santa Fé, Playa, La Habana 19108, Cuba; 4 Institut de Biotecnologia i Biomedicina and Departament de Bioquimica i Biologia Molecular, Universitat Autònoma de Barcelona, Bellaterra, 08193 Barcelona, Spain

**Keywords:** *Moringa oleifera*, protease, plant protease inhibitor, phytocystatin, papain inhibitor, antibacterial, anticoagulant, bioactive compound

## Abstract

Plant cystatins (or phytocystatins) comprise a large superfamily of natural bioactive small proteins that typically act as protein inhibitors of papain-like cysteine proteases. In this report, we present the purification and characterization of the first phytocystatin isolated from *Moringa oleifera* (MoPI). MoPI has a molecular mass of 19 kDa and showed an extraordinary physicochemical stability against acidic pHs and high temperatures. Our findings also revealed that MoPI is one of the most potent cysteine protease inhibitors reported to date, with *K*i and IC_50_ values of 2.1 nM and 5.7 nM, respectively. More interestingly, MoPI presents a strong antimicrobial activity against human pathogens such as *Enterococcus faecalis* and *Staphylococcus aureus*. In addition, MoPI also showed important anticoagulant activity, which is an unprecedented property for this family of protease inhibitors. These results highlight the pharmaceutical potential of this plant and its derived bioactive molecules.

## 1. Introduction

*Moringa oleifera* (*M. oleifera*) is a plant native to India that grows in the tropical and subtropical regions of the world. Due to its high nutritional properties, the whole plant is typically used for nutritional or commercial purposes. It is worth mentioning that different parts of this tree are applied as food to combat malnutrition, especially among infants and breastfeeding woman in many developing countries, including India, Pakistan, the Philippines, Hawaii and many regions of Africa [1].

In addition to its nutritional value, *M. oleifera* has an important medicinal and pharmacological potential. Recent studies have shown that extracts of this plant possess strong antioxidant, anticancer, anti-inflammatory and antidiabetic properties [2]. Furthermore, evidence suggests that almost all parts of the plant exhibit antibacterial activity [3]. In many cases, such antibacterial activity has been associated with the presence of small chemical compounds such as glucosinolates (β-thioglucoside N-hydroxysulfates), isothiocyanates, organic carbamates, chalcone-oxazolidinone hybrids and thiocarbamate [4]. Although extensive research has been carried out with *M. oleifera* extracts, little is known regarding the presence of protease inhibitors in this plant and their role as antibacterial agents.

Cysteine proteases (CPs) are among the most widely distributed and relevant proteases. They are present in almost all living organisms and participate in a variety of biological processes [5]. It has been reported that the imbalance in the activity of endogenous CPs can lead to numerous pathologies such as rheumatoid arthritis, multiple sclerosis, neurological disorders, tumours, and osteoporosis [6]. CPs produced by bacteria, viruses, and parasites are also considered important factors in the development of many human diseases such as paradontosis [7], malaria, Chagas disease and schistosomiasis [8,9], among others. Special mention deserves the role of the papain-like cysteine protease (PCPs) from the SARS-CoV-2, which is required for the processing of viral polyproteins during viral spread and to evade the innate immunity of the host [10]. From a more general point of view, PCPs play essential roles in growth, cell differentiation, signalling and host invasion in pathogenic organisms and frequently act as a virulence factor by attacking the host’s immune system [10,11,12,13]. Thus, fine control of the proteolytic activity is essential for the proper functioning of whole cells and organisms. In fact, this is achieved at many levels, from the regulation of protein expression, secretion and maturation (through the specific cleavage of the proenzyme) to the blocking of its activity by inhibition by specific protease inhibitors [14].

Cystatins (or phytocystatins) are a family of structurally related proteins with molecular masses in the range of 12–23 kDa that function as CPs inhibitors [15]. Previous studies suggested that phytocystatins isolated from corn, barley, tomato, rice, papaya, etc., [16,17,18] can inhibit insect CPs in vitro [19,20,21]. On the other hand, phytocystatins isolated from chestnut, sugar cane, carnation, barley, taro, strawberry, wheat, cocoa, amaranth and sesame showed strong inhibitory activity against broad-range fungal pathogens [22]. However, despite all these previous studies, there is only one known cystatin that specifically inhibits the growth of bacteria. This protein has been isolated from kiwi and is capable of blocking the growth of *Agrobacterium tumefasciens*, *Burkholderia cepacia*, and *Erwinia carotovora* [23].

Over the past years, *M. oleifera* has shown great nutritional and pharmacological potential. However, previous research has been focused on evaluating the activity of crude extracts and the identity and biological role of its bioactive compounds remains elusive. In the present study, we report the isolation, purification and biochemical characterization of a novel papain inhibitor obtained from *M. oleifera* seeds. Herein, we also studied the antibacterial properties of the isolated bioactive inhibitor, which was assayed against a panel with nine different bacterial strains. More importantly, anticoagulant activity studies results indicated that MoPI is a pharmacologically active anticoagulant molecule. Taken together, the activities and distinctive properties of this novel inhibitor represent the first report carried out for *M. oleifera* phytocystatins and suggests direct applicability of this bioactive molecule for pharmaceutical and/or biotechnological applications.

## 2. Materials and Methods

### 2.1. Materials

The seeds of *M. oleifera* were hand-collected from local trees near La Coronela neighborhood, La Habana, Cuba. The seeds were washed and stored at −80 °C until use. Coomassie Blue G-250, *N*,*N*,*N*′*,N*′-tetramethylethylenediamine (TEMED), sodium chloride, tris (hydroxymethyl) aminomethane, sodium dodecyl sulphate (SDS), β-mercaptoethanol (βME), bovine serum albumin (BSA), l-pyroglutamyl-l-phenylalanyl-l-leucine-p-nitroanilide (PFLNA), Trypsin, 4-nitrophenol-α-d-glucopyranoside (PNPG) and α-glucosidase were purchased from Sigma-Aldrich (San Luis, MO, USA). Glyoxyl-agarose was delivered from FlukaTM. All other chemicals used in this work were of analytical grade and purchased from Sigma-Aldrich (San Luis, MO, USA) (unless otherwise specified).

### 2.2. Crude Extract Preparation

*M. oleifera* seeds (30 g) were washed with distilled water and dried at room temperature during 16–20 h. The seeds were ground using a blender with the addition of 450 mL of 0.01 M phosphate buffer, pH 7.4 in an ice bath to avoid possible protein denaturation. After incubation for 2 h at room temperature, the mixture was filtered with gauze, and the insoluble material was removed by centrifugation for 30 min at 7000× *g* at 4 °C. The clarified supernatant (from now on: MoCE) was collected and immediately frozen at −20 °C until analysis. The total protein content was determined by the Bradford’s assay [24], as described by Cotabarren et al. [25] using bovine serum albumin (BSA) as standard (0.1 mg/mL). Papain inhibitory activity was determined as described in Section 2.4.

### 2.3. Identification and Purification of MoPI

#### 2.3.1. Partial Purification by Heat Treatment

According to our previous studies, protease inhibitors present high physicochemical stability with minimal loss of inhibitory activity [26]. Accordingly, in the first purification step, the crude extract was subjected to 100 °C for 15, 60, and 120 min. Afterward, the samples were cooled at room temperature, and the thermally denatured proteins were removed by centrifugation for 30 min at 7000× *g* and 4 °C. The total protein content and the inhibitory activity of the non-treated crude extract and heat-treated samples were determined. Each obtained sample was called MoHT15, MoHT60 and MoHT120, in accordance to the incubation time.

#### 2.3.2. Affinity Chromatography

A sample of 150 mL of MoHT15 was loaded onto a papain-glyoxyl-agarose column prepared in-house following the method of Obregón and colleagues [27] (1.5 × 12 cm) connected to an Äkta-Purifier (GE Healthcare, Chicago, IL, USA) previously equilibrated with 0.01M phosphate buffer, pH 5.5–considering the optimal pH for the papain-inhibitor interaction–. After complete removal of the non-retained proteins with equilibration buffer, affiliated proteins were eluted generating a sudden pH change by the addition of 0.01 M HCl, pH 2.5 at a flow rate of 0.7 mL/min. The eluted fractions were immediately neutralized by adding 0.1 M NaOH. The purified papain inhibitor was named MoPI (*M. oleifera* papain inhibitor). Papain inhibitory activity and protein quantification were determined as previously described. Samples were analysed by SDS-PAGE as described by Cotabarren et al. [25]

### 2.4. Enzymatic Analysis of the Inhibitory Activity against Papain 

Papain inhibitory activity was determined by using the substrate L-pyroglutamyl-L-phenylalanyl-L-leucine-p-nitroanilide (PFLNA). The hydrolysis of PFLNA was monitored by measuring the increase of the absorbance at 410 nm at 37 °C every minute for 10 min [27]. Reaction volumes were adapted to a 96-well plate with a final volume of 200 µL. The inhibition of papain activity caused a decrease in the hydrolysis rate of the substrate, which resulted in the attenuation of the hydrolysis slope. The inhibitory activity was estimated as the residual proteolytic activity in the presence of the inhibitor and expressed as a percentage of inhibition in comparison with the control assay (in the absence of the inhibitor). In the case of heat-treated samples, the volume used in each assay was 20 µL.

One papain inhibitory unit (1 PIU) was defined as the decrease in 0.01 unit of absorbance at 410 nm per 10 min assay, at 37 °C. The inhibitory constant (*K*i) and the IC_50_ (defined as the inhibitor concentration required for half-activity of the enzyme) were calculated according to the protocol described by Tellechea and colleagues [28], modified for papain inhibition. All measurements were performed in triplicate.

### 2.5. Biological Assays

#### 2.5.1. Anticoagulant Activity

Anticoagulant activity of MoPI was evaluated by determining prothrombin time (PT) and the time of activated partial thromboplastin (aPTT) using a Coatron M1 coagulometer (TECO, Neufahrn, Germany). In both cases, a pool of blood plasmas from the mixture, in equal parts, of 5 healthy individuals, maintained at 37 °C with 3.8% sodium citrate (ratio sample: anticoagulant 9:1) was used as a sample (from now on: PBP).

For the PT test, the commercial Soluplastin reactive (Wiener Lab.) was employed. Initially, equal parts of the PBP sample and the papain inhibitor (12.5 µg/mL) were incubated for 2 min at 37 °C, then 50 µL of Soluplastin were added to 25 µL of this mixture and checked for the coagulation time. For the aPTT test, 25 µL of aPTT (Wiener Lab.) were added to an equal volume of PBP-inhibitor mixture (previously incubated for 2 min at 37 °C). After 2 min incubation at 37 °C, 25 µL of 50 mM CaCl_2_ was added to initiate the coagulation time determination. For both assays, measurements were carried out in triplicate and appropriate controls were achieved.

#### 2.5.2. Antimicrobial Activity

In order to determine the inhibitory capacity on the growth of various bacterial strains by the inhibitor, the agar diffusion assay based on the Kirby-Bauer test was performed with slight modifications [29]. Initially, the pre-inoculums of different pathogenic microorganisms (i.e., *Citrobacter amalonaticus*, *Enterobacter cloacae*, *Enterococcus faecalis*, *Escherichia coli*, *Klebsiella pneumoniae*, *Proteus vulgaris*, *Pseudomonas aeruginosa*, *Salmonella typhimurium* and *Staphylococcus aureus*) were grown at 37 °C for 18–24 h and the turbidity of the bacterial suspension was adjusted with a physiological solution to 0.5 McFarland scale (1.5 × 108 UFC/mL). The inoculums were plated on Müller Hinton agar plates, in which a drop of 10 µL inhibitor was placed. After the drop placed in the plate was dry, the plates were incubated at 37 °C for 24 h. Then, the diameters of the bacterial growth inhibition halos (in millimetres) were measured. The tests were conducted by triplicate, including the respective controls.

### 2.6. Statistical Analysis

Statistical analyses (ANOVA) were performed with GraphPad Prism (v6.03, GraphPad Sofware Inc.: San Diego, CA, USA, 2012). Significant differences between the means of the parameters were determined by Tukey’s post-hoc test (*p* < 0.05).

## 3. Results and Discussion

### 3.1. Isolation and Purification of MoPI

Moringa seeds are known to be rich sources of lipids (31%), and their elimination leads to a high protein expeller (19%), generating an interesting product for human nutrition. As described in Section 2.2, we prepared a crude extract of *M. oleifera* seeds (MoCE). The protein concentration of the sample was 3.51 ± 0.03 mg/mL (see Table 1), which is in agreement with previous reports [30]. Next, we aimed to investigate the papain inhibitory activity of the crude extract. This initial examination showed that small amounts of MoCE to the reaction tube produce a drastic decrease in the papain activity, which is consistent with the presence of inhibitory molecules in the sample. A more detailed dose-response analysis showed that MoCE strongly inhibits papain with an IC_50_ value of 0.025 µg/mL.

**Figure 1:**
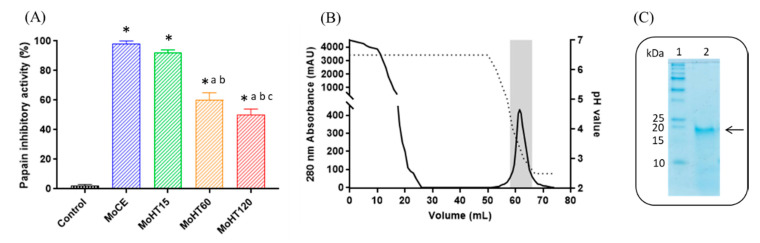
Isolation and purification of *M. oleifera* papain inhibitor (MoPI) from the crude extract (MoCE). (**A**) Papain inhibitory activity (%) in the presence of the different *M. oleifera* heat-treated samples. MoCE: *M. oleifera* crude extract, MoHT15-120: *M. oleifera* crude extract after 100 °C heat treatment for 15, 60 and 120 min. * *p* < 0.05, compared to control; ^a^ *p* < 0.05 compared to MoCE; ^b^ *p* < 0.05 compared to MoHT15; ^c^ *p* < 0.05 compared to MoHT60 (one-way ANOVA and Tukey´s multiple comparison test). (**B**) Purification of *M. oleifera* papain inhibitor by affinity chromatography on immobilized papain. Fractions with papain inhibitory activity (gray area) were pooled and named MoPI. (**C**) Electrophoresis (SDS-PAGE, 12% *v*/*v*) of the *M. oleifera* papain inhibitor (MoPI). Lane 1: Low range molecular mass marker; Lane 2: Purified *M. oleifera* papain inhibitor. The arrow indicates the location of the inhibitor.

A number of plant protease inhibitors (PIs) have been purified and found to be stable and active at high temperatures [26,31]. For this reason, we incubated the MoCE at 100 °C for 15, 60 and 120 min as an initial purification step. As observed in Table 1, the heat treatment at 100 °C produced a decrease in protein content of 50–60% with respect to the raw starting crude extract. Papain inhibitory activity was then determined in the samples corresponding to each heat treatment (MoHT15, MoHT60 and MoHT120). As shown in Figure 1A, the inhibitory activity against papain was maintained even after 120 min of incubation, suggesting an extraordinary thermostability of the inhibitor. It is worth mentioning that this property is very interesting for most biotechnological applications, behind an added value for their commercial exploitation [32].

In addition to the thermal stability, we also evaluated the effect of the temperature on the stability of the inhibitor at two extreme pH values (pH 2 and 9). Surprisingly, after 60 min of incubation, we were able to recover 100% of the inhibitory activity against papain. This unusual physicochemical stability is a remarkable feature for phytocystatins. So far, there is only one previous report describing a papain inhibitor isolated from *Vigna unguiculata* seeds that presented both thermal stability and a similar tolerance to a wide range of pHs [33].

As shown in Table 1 and Figure 1A, MoHT15 is the sample with a better relationship between conservation of activity/elimination of soluble non-inhibitory proteins. Moreover, this thermal treatment represents a simple and crucial purification step to achieve a partial purification of the inhibitor. MoPI was purified from the MoHT15 fraction after performing a high-speed centrifugation step (see Materials and Methods section for experimental details). After centrifugation, the supernatant containing the inhibitor was further purified by affinity chromatography using a special support based on glyoxyl-agarose containing covalently immobilized papain. After purification by affinity chromatography, the papain inhibitory activity of the eluted fractions was determined. As shown in Figure 1B, the purified inhibitor eluted as a single peak that contained a protein concentration of 0.16 ± 0.01 mg/mL and specific inhibitory activity of 10.81 PIU/mg (see Table 2). After the different purification steps, the resultant MoPI showed an apparent molecular weight of 19 kDa (Figure 1C). This size is in agreement with the size of other phytocystatins previously isolated from plants (i.e., garlic phytocystatin [34], Barley protease inhibitor and *Vigna unguiculata* cysteine inhibitor [33].

### 3.2. Inhibition Kinetics

**Figure 2:**
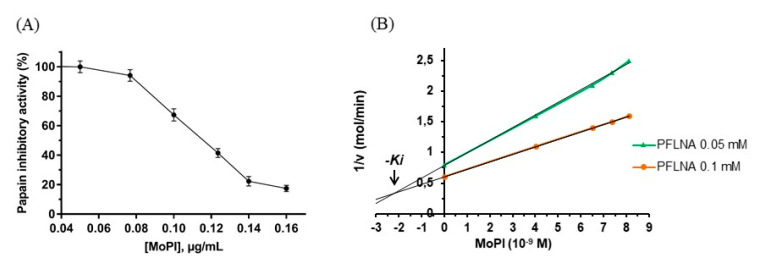
Kinetic studies on *M. oleifera* papain inhibitor. (**A**) Dose response curve for the half maximal inhibitory concentration (IC_50_) determination. (**B**) Dixon plot (1/v vs. [I]) for identification of the *Ki*. Each point represents the mean of three estimates.

Kinetic studies of MoPI inhibition activity were carried out following the protocol described by Tellechea and colleagues [28]. Analysis of the data revealed that MoPI has an IC_50_ value of 0.11 µg/mL (5.7 × 10^−9^ M, Figure 2A) and a *K*i value of 2.1 × 10^−9^ M (Figure 2B), indicating that MoPI is a potent papain inhibitor. In comparison to other high thermostable protease inhibitors previously reported in the literature [26], MoPI presents one of the lowest IC_50_ values (see Table 3). MoPI also presents a *K*i that is significantly lower in comparison to other thermostable PIs; i.e., papaya proteinase inhibitor (*K*i = 3 × 10^−7^ M; [35]), *Cajanus cajan* proteinase inhibitor (2.72 × 10^−7^ M, [36]), *Albizia amara* protease inhibitor (1.24 × 10^−8^ M, [37]) and garlic protease inhibitor (8.5 × 10^−8^ M, [34]).

Interestingly, while most of the phytocystatins maintains only 10–25% of their inhibitory capacity after 80–90 °C incubation, MoPI preserves total inhibitory capacity after 15 min incubation at 100 °C, being one of the most stable phytocystatins reported to date (see Table 3). Only VuCys1 has a higher thermostability after 60 min incubation at 100 °C. However, the inhibitory kinetics of this inhibitor is still unknown. It is important to mention that although the phytocystatins included in Table 3 were classified by their authors as highly thermostable PIs, we reported on a recently published article [26] that such phytocystatins do not fall within the super stable inhibitors. For this reason, the current stability data, the IC_50_ and *K*i position MoPI as one of the phytocystatins with the greatest potential for biotechnological applications. Several studies aimed at characterizing the dependence of the thermal stability of plant PIs on pH established the optimal conditions for the application of these proteins to biotechnology involved in the development of transgenic crops resistant to insect pests [38] or in the pharmaceutical industry as specific inhibitors of pathogens such as fungi and bacteria [39].

### 3.3. Anticoagulant Activity

Thrombotic events due to blood clotting are known to pose a serious problem in cardiovascular disease [45]. Although heparin (a protease inhibitor widely used to delay blood clotting time) has been widely used for this purpose, its continued use often results in the development of thrombocytopenia and immune response [46]. Furthermore, other anticoagulant drugs, such as aspirin and clopidogrel, may lead to serious side effects [47]. In this context, only serine protease inhibitors have been studied as potential antithrombotic agents [48,49,50]. Discovery of new PIs with inhibitory activity of the coagulation cascade would result in an alternative strategy against thrombosis.

The anticoagulant activity of MoPI was tested by determining the activated partial thromboplastin time and the time of prothrombin (Table 4). The concentration of inhibitor evaluated (12.5 µg/mL) produced a significant increase of 25% in the time of activated partial thromboplastin compared to the control sample. This result suggests a potential application of the *M. oleifera* papain inhibitor as an anticoagulant agent of natural origin as an alternative to conventional anticoagulant agents. Regarding the behaviour of MoPI for the extrinsic route (see Prothrombin time in Table 4), the values obtained for the corresponding test were very similar to those of the control sample; therefore, no significant increase in coagulation time was observed. 

### 3.4. Antimicrobial Activity 

The antimicrobial activity of the crude extract (MoCE) and heat-treated sample (MoHT15) were evaluated in order to establish whether the powerful papain inhibitor from *M. oleifera* is able to inhibit bacterial growth. As observed in Table 5, our results confirmed the findings from Bancessi and colleagues [3] that reported a strong antimicrobial activity of Moringa seed extracts against *E. coli*, *E. faecalis*, *S. aureaus*, *E. cloacae*, *Proteus*, *K. pneumoniae*. More interestingly, the heat-treated sample showed a strong inhibitory effect against the growth of *E. faecalis* and *S. aureaus*. A similar profile of inhibition was observed for the purified inhibitor MoPI, suggesting that this papain inhibitor has strong antibacterial properties against these two bacterial strains. It should be noted that the tests were performed using a relatively low sample concentration. 

As discussed above, the only known phytocystatin with antibacterial activity is a kiwi cystatin that inhibits the growth of *Agrobacterium tumefaciens*, *Burkholderia cepacia,* and *Erwinia carotovora* [23]. This study represents new evidence of the antimicrobial role of phytocystatins, expanding their possible biomedical applications. Genetically engineered cystatins could be generated and act as potent nutraceuticals in the development of a food product designed from a “plant derivative” that allows preventing the infection of pathogens in the human digestive system. So far there is no evidence that cysteine proteases (absent in the human intestinal system) have any role (as compared to serine proteases) in the degradation of human food. Therefore, plant cystatins would have appropriate characteristics to be used as pharmaceutical products and/or to be incorporated as nutraceuticals due to their antimicrobial and antiviral properties, since they can only interact with microbial or viral cysteine proteases in the human intestine and not with cysteine proteases in the human digestive system [51].

It is known that, with the aim of getting the properties of Moringa to the consumer, studies are being carried out with different foods, mainly meat products and breads, in which Moringa (leaf, seed, extracts, etc.) is incorporated as an ingredient. In meat products, it is used as a preservative and antioxidant additive with very good results without affecting the sensory characteristics of the final product. In the bakery sector, the objective is usually the nutritional enrichment of the food. It would be very interesting to incorporate Moringa seed extracts as a food additive, substituting different preservatives and chemical antioxidants, and at the same time its use in the preparation of highly nutritious basic products, ideal for certain population groups in greater risk of malnutrition. There are indications that the industry is gearing up for a large investment in “designer foods” with benefits for humans. Such foods, which could ultimately decrease demand for drugs, could also help control disease and disorders often associated with a change in lifestyle.

## 4. Conclusions

Herein, we report the isolation and purification of the first papain inhibitor from *M. oleifera* seeds. This novel bioactive molecule demonstrated a great physicochemical stability at high temperatures and extreme pHs. In addition, MoPI showed a surprising inhibitory activity against papain with *K*i and IC_50_ values in the nanomolar range, making this inhibitor one of the most powerful phytocystatins found to date. On the other hand, the growth inhibitory capacity was determined on various strains of pathogenic bacteria, demonstrating a strong antimicrobial effect of this inhibitor against *Enterococcus faecalis* and *Staphylococcus aureus* strains. Moreover, we also anticipate that MoPI isolated from *M. oleifera* has intrinsic anticoagulant activity against the intrinsic pathway of the coagulation cascade. Taken together, these properties position this novel molecule as a potential natural antibacterial agent suitable for biotechnological and pharmaceutical applications. This research also contributes to the knowledge of unprecedented characteristics in this type of inhibitor, being the first cysteine protease inhibitor of vegetable origin with this particular combination of biological activities.

## Figures and Tables

**Table 1:** Concentration for *M. oleifera* crude extract and heat-treated samples.

Sample	Total Protein Content (mg/mL) ^a^
*M. oleifera* crude extract (MoCE)	3.51 ± 0.03
*M. oleifera* crude extract after 100 °C heat treatment for 15 min (MoHT15)	1.73 ± 0.05
*M. oleifera* crude extract after 100 °C heat treatment for 60 min (MoHT60)	1.66 ± 0.06
*M. oleifera* crude extract after 100 °C heat treatment for 120 min (MoHT120)	1.5 ± 0.04

^a^ Values are the mean ± standard deviation (n = 3).

**Table 2:** Purification steps of MoPI from *Moringa oleifera* seeds.

Purification Step	Total Protein Amount (mg)	Total Inhibitory Activity (PIU) ^a^	Specific Inhibitory Activity (PIU/mg)	Purity (fold) ^b^	Yield (%) ^c^
Crude extract	561.6 ± 4.8	42.5 ± 6.7	0.07 ± 0.01	1.0	100
100 °C heat treatment	259.5 ± 7.5	39.8 ± 4.3	0.15 ± 0.01	2.1	93.7
Affinity chromatography	1.3 ± 0.1	14.1 ± 2.4	10.81 ± 1.31	154.4	33.2

^a^ One papain inhibitory unit (1 PIU) was defined as the amount of inhibitor that decreased absorbance at 410 nm by 0.1 under the assay conditions. ^b^ The purification index (Purity) was calculated as the ratio between the specific inhibitory activity determined after each purification step as compared to the initial inhibitory activity present in the crude extract. ^c^ Yield of inhibitory activity after each purification step compared to the crude extract (%).

**Table 3:** Thermostable phytocystatins isolated in the last 20 years.

Inhibitor	Plant Name	Origin	*Ki* (M)	IC_50_	MW (kDa)	Temp (°C)	Time (min)	RIA (%)	Ref.
GPC	*Allium sativum*	Cloves	8.5 × 10^−8^	N/D	12.5	90	30	10	[34]
VuCys1 and VuCys2	*Vigna unguiculata*	Recombinant (Leaves)	N/D	N/D	10.7 (1) 21.9 (2)	100	60	VuCys1: 100 VuCys2: 90	[33]
YMP	*Brassica alba*	Seeds	3.1 × 10^−7^	9.0 × 10^−7^	26.4	90	30	10	[15]
Almond cystatin	*Prunus dulcis*	Fruits	4.54 × 10^−8^	N/D	63.4	90	60	15	[40]
Mustard TPI	*Brassica juncea*	Seeds	1.0 × 10^−7^	N/D	18.1	90	30	25	[41]
SBPC	*Gicine max*	Soybean	3.59 × 10^−6^	N/D	19	80	30	15	[42]
PMC I and PMC II	*Phaseolus mungo*	Seeds	N/D	N/D	19.1 (I) 17.5 (II)	90	30	80	[43]
CICPI	*Cassia leiandra*	Seeds	4.1 × 10^−7^	8.5 × 10^−7^	18.3	100	20	55	[44]
MoPI	*Moringa oleifera*	Seeds	2.1 × 10^−9^	5.7 × 10^−9^	19	100	15 60	90 60	

Abbreviations: *K*i: inhibitory constant; IC_50_: amount of inhibitor needed for 50% papain inhibition; MW: molecular weight; Temp: Temperature; Time: Incubation time; RIA: Residual Inhibitory Activity Ref.: References.

**Table 4:** Anticoagulant activity of MoPI on activated partial thromboplastin and prothrombin.

Sample	Prothrombin Time (seg) ^a^	Activated Partial Thromboplastin Time (seg) ^a^
Control	18.6 ± 0.7	44.4 ± 3.1
MoPI	18.1 ± 0.7	60.6 ± 0.9 *

^a^ Values are the mean ± standard deviation (n = 3). * *p* < 0.05, compared to control (two-tailed *t*-test).

**Table 5:** Evaluation of the antibacterial activity of *M. oleifera* crude extract (MoCE), heat-treated sample (MoHT15) and the purified inhibitor (MoPI).

Pathogenic Bacteria	MoCE (175 µg)	MoHT15 (85 µg)	MoPI (24 µg)
*Citrobacter amalonaticus* CIPROVE	-	-	-
*Enterobacter cloacae* CIPROVE	+	-	-
*Enterococcus faecalis* ATCC 29212	++	++	++
*Escherichia coli* ATCC 25922	+	-	-
*Klebsiella pneumoniae* ATCC 700603	+	-	-
*Proteus vulgaris* CIPROVE	+	-	-
*Pseudomonas aeruginosa* ATCC 27853	-	-	-
*Salmonella typhimurium* CIPROVE	-	-	-
*Staphylococcus aureus* ATCC 29213	+++	+++	+

ATCC: American Type Culture Collection; CIPROVE: Culture collection of the CIPROVE, Facultad de Ciencias Exactas, Universidad Nacional de La Plata. Inhibition zones: (+++) inhibition zone higher than 7 mm; (++) inhibition zone of 6 mm; (+) inhibition zone of 5 mm; - (no inhibition).

## Data Availability

Not applicable.
